# Relationships between genome-wide R-loop distribution and classes of recurrent DNA breaks in neural stem/progenitor cells

**DOI:** 10.1038/s41598-022-17452-0

**Published:** 2022-08-04

**Authors:** Supawat Thongthip, Annika Carlson, Magdalena P. Crossley, Bjoern Schwer

**Affiliations:** 1 The Eli and Edythe Broad Center of Regeneration Medicine and Stem Cell Research, University of California, San Francisco, CA USA; 2 Bakar Aging Research Institute, University of California, San Francisco, CA USA; 3 Kavli Institute for Fundamental Neuroscience, University of California, San Francisco, CA USA; 4 Weill Institute for Neuroscience, University of California, San Francisco, CA USA; 5 Department of Neurological Surgery, University of California, San Francisco, CA USA; 6 Department of Chemical and Systems Biology, Stanford University School of Medicine, Stanford, CA USA

**Keywords:** DNA damage and repair, Ageing, Embryonic stem cells, Neural stem cells, Genomic instability

## Abstract

Recent studies revealed classes of recurrent DNA double-strand breaks (DSBs) in neural stem/progenitor cells, including transcription-associated, promoter-proximal breaks and recurrent DSB clusters in late-replicating, long neural genes that may give rise to somatic brain mosaicism. The mechanistic factors promoting these different classes of DSBs in neural stem/progenitor cells are not understood. Here, we elucidated the genome-wide landscape of RNA:DNA hybrid structures called “R-loops” in primary neural stem/progenitor cells undergoing aphidicolin-induced, mild replication stress to assess the potential contribution of R-loops to the different, recurrent classes of DNA break “hotspots”. We find that R-loops in neural stem/progenitor cells undergoing mild replication stress are present primarily in early-replicating, transcribed regions and in genes with promoter GC skew that are associated with cell lineage-specific processes. Surprisingly, most long, neural genes that form recurrent DSB clusters do not show R-loop formation under conditions of mild replication stress. Our findings are consistent with a role of R-loop-associated processes in promoter-proximal DNA break formation in highly transcribed, early replicating regions but suggest that R-loops do not drive replication stress-induced, recurrent DSB cluster formation in most long, neural genes.

## Introduction

Genome stability is important for cellular function but the genome of somatic cells shows much more plasticity than previously thought^1^. In mammals, somatic genomic alterations have traditionally been viewed primarily as a cause of cancer but are now emerging as drivers of organismal aging and brain disorders^1–5^. Somatic genomic alterations can arise from DNA double-strand breaks (DSBs) formed during normal cellular processes such as DNA replication and transcription. Mammalian cells use evolutionarily-conserved mechanisms to repair DSBs and maintain genome integrity^1^. In the nervous system, persistent DSBs caused by deficient repair can result in microcephaly, neurodegenerative disorders, and brain tumorigenesis^5^.

Recent studies have identified several recurrent classes of DSBs in human and murine neural stem/progenitor cells (NSPCs) via high-throughput genome-wide translocation sequencing (HTGTS)^4,6–9^. Such classes include widespread, low-level DSBs, transcription start site (TSS)-proximal DSBs, and recurrent DSB clusters (RDCs) in long neural genes^1,4,6–9^. Most RDCs in transcribed, long neural genes occur in gene bodies and are not associated with TSSs^6–9^, indicating that distinct mechanisms of DSB generation account for the different classes of DSBs in NSPCs.

Given the frequency and potential functional implications of RDCs in NSPCs, it is important to elucidate their mechanistic causes. In that regard, collisions of the transcription and replication machineries can cause genomic instability in mammalian cells^10^. Indeed, prior studies of RDCs and copy number variations (CNVs)^4,6,11^ suggest that formation of the underlying DSBs may involve transcription/replication collisions in late-replicating regions^1,11,12^. Consistent with that notion, RDCs in neural progenitor cells form within genes and are enhanced by DNA replication stress^1,4,6^. However, because the majority of long, transcribed, and late-replicating genes do not contain RDCs^6^, additional factors must influence RDC formation.

To define such mechanistic factors, we considered whether transcription-related processes may affect RDC formation. Specifically, we asked whether RNA:DNA hybrid structures known as “R-loops” promote recurrent DNA breaks in NSPCs. R-loops consist of an RNA:DNA hybrid and the corresponding, displaced single-stranded DNA, thus forming a three-stranded nucleic acid structure^13^. Although R-loops have been known for over 50 years, their biological roles are still unclear. R-loops are emerging as important non-B DNA structures that form in transcribed loci^13,14^. Traditionally, R-loops have been viewed as obstacles impeding ongoing transcription that need to be removed, and as sources of DNA damage that can induce single- and double-strand breaks and genomic instability^14–18^.

How R-loops cause genomic instability is still unclear^13^. Pausing of transcription—which occurs when RNA polymerase II (RNAPII) progression is hindered—can induce RNAPII backtracking, which may form R-loops ahead of the backtracked RNAPII^19,20^. Thus, formation of R-loops could be an important contributing factor for the generation of both TSS-proximal DSBs and RDCs within long neural genes in NSPCs. To address this, we elucidated the genome-wide landscape of R-loop formation in NSPCs and assessed functional implications and relationships between these nucleic acid structures and classes of DSBs in NSPCs under conditions of mild replication stress.

## Results

### Genome-wide mapping of R-loops in NSPCs

**Figure 1:**
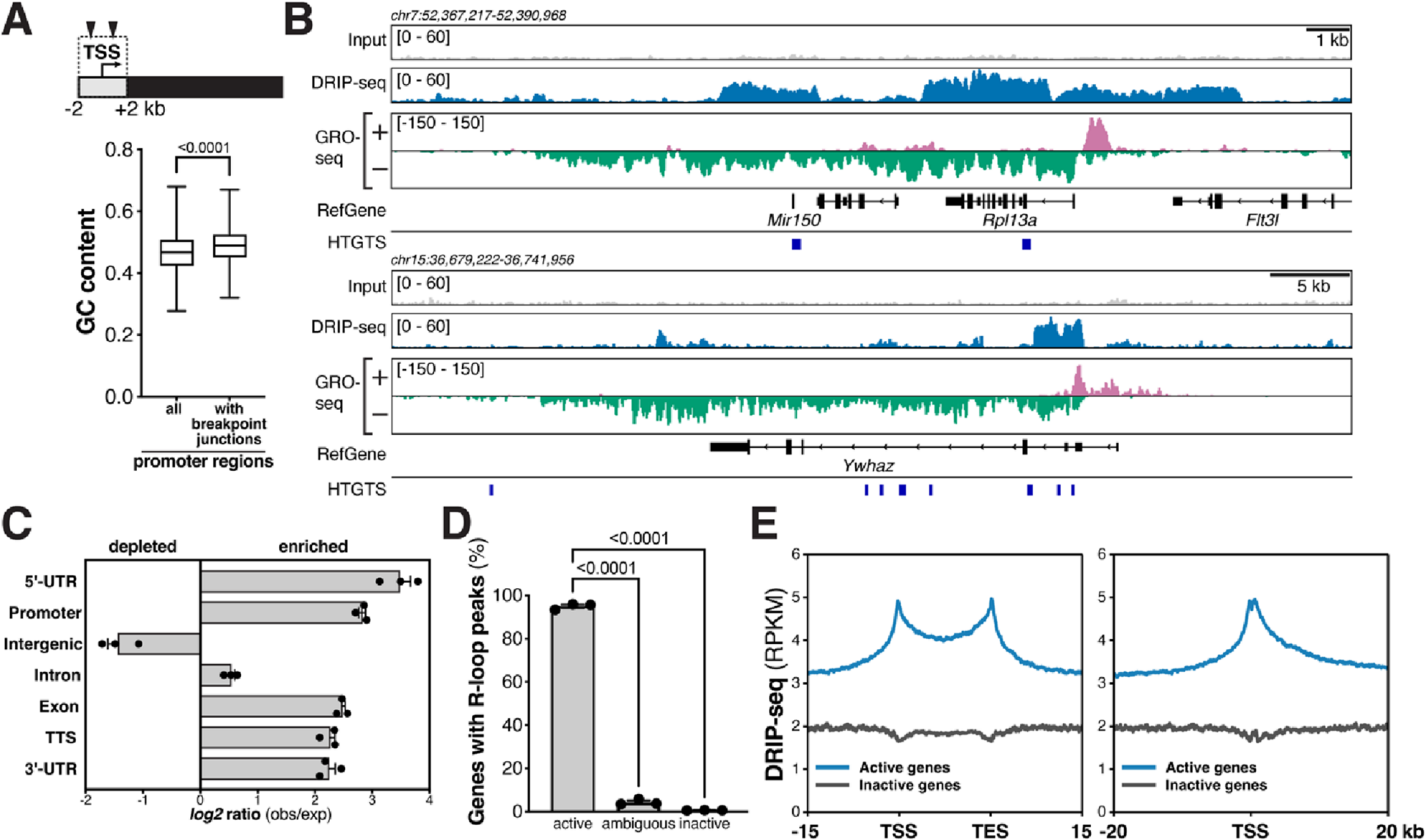
Elucidation of R-loops in neural stem/progenitor cells. (**A**) *Top*, illustration of genes with DNA breakpoints in the promoter region, defined as the two kilobase (kb) region surrounding the transcription start site (TSS). Triangles illustrate HTGTS breakpoint junctions. *Bottom*, box-and-whiskers plot showing fractional GC content in promoter regions of all NCBI37/mm9 RefSeq genes (*n* = 22,735) and actively transcribed genes with HTGTS junctions within two kb of the TSS in NSPCs (*n* = 2332). Whiskers show minimum and maximum values; upper and lower box edges correspond to the 25th and 75th percentile; horizontal line indicates the median. *P* < 0.0001, Mann–Whitney U test. (**B**) Visualization of reads per kilobase per million (RPKM)-normalized DRIP-seq signal in input controls and DRIP samples over the indicated genomic regions. Combined signal from nine DRIP samples and matching input controls from three biological replicates is plotted. RPKM-normalized GRO-seq signal is plotted to show transcription. Location of RefSeq genes and position of DSB junctions detected by high-throughput, genome-wide, translocation sequencing (HTGTS) of aphidicolin-treated NSPCs is shown. (**C**) Genomic distribution of NSPC R-loop peaks across the indicated genome annotations compared to the expected genomic distribution. (**D**) Transcriptional status of genes with R-loop peaks in NSPCs. Data in (**C**) and (**D**) represent mean ±  S.E.M. from three independent DRIP-seq experiments performed on biological replicates. *P* values were determined by one-way ANOVA with Tukey’s post hoc correction for multiple comparisons. (**E**) *Left*, metaplot analysis of RPKM-normalized, raw DRIP-seq signal across active (blue, *n* = 15,528) and inactive (dark gray, *n* = 3246) genes in NSPCs reveals enrichment at TSSs, gene bodies, and transcription end sites (TESs). *Right,* RPKM-normalized, raw DRIP-seq signal around the TSSs of active and inactive genes in NSPCs.

Several classes of recurrent DSBs occur in mouse and human NSPCs, including DSB breakpoint clusters in long, transcribed and late-replicating genes and around transcriptional start sites^4,6–9^. To elucidate potential mechanistic factors involved in the formation of the different classes of recurrent DSBs, we assessed the genomic features of regions surrounding breakpoint junctions in NSPCs. We noted that the promoter regions—defined as regions ± 2 kb of the TSS—of active genes with HTGTS breakpoint junctions^6,7,9^ showed a significantly higher content of guanine/cytosine (GC) nucleotides (Fig. 1A). This prompted us to consider the role of R-loops in the formation of DSBs in NSPCs, given that regions with high G density in the non-template strand are prone to R-loop formation^16^. Moreover, R-loop formation has been implicated as a cause of genomic fragility in a subset of long human genes^12^, suggesting a potential involvement in the formation of recurrent DSB clusters in long, neural genes (RDC-genes)^4,6–8^.

To directly assess the potential role of R-loops in the formation of TSS-proximal DSBs or recurrent DSB clusters in long neural genes, we set out to elucidate the genomic landscape of R-loops in NSPCs under the same conditions of aphidicolin (APH)-induced, mild replication stress that we had previously used to identify recurrent classes of DSBs in this cell type^6,9^. Reliable, high-resolution mapping of R-loops has become possible by multiple approaches, including “DNA:RNA immunoprecipitation with deep sequencing” (DRIP-seq)^21–24^. This approach relies on the S9.6 monoclonal antibody that specifically binds RNA:DNA hybrids and allows quantitative recovery of R-loops in conjunction with the high-resolution mapping capability of next-generation sequencing^21^. We first validated the S9.6 antibody and DRIP approach by performing dot blots and DRIP-quantitative PCR (DRIP-qPCR) assays, using established positive and negative controls (Fig. S1A and B). Next, we performed DRIP-seq in primary NSPCs isolated from postnatal day 7 mice in the presence of mild, aphidicolin-induced replication stress as described^6,9^. To assess the quality of our R-loop mapping in NSPCs, we visualized raw DRIP-seq signal over the "gold standard" *Rpl13a* and the *Ywhaz* gene promoter regions^21^. Consistent with previous reports in human cells^21^, we detected robust DRIP-seq signal over these regions in mouse NSPCs (Fig. 1B). Visual comparison of raw DRIP-seq signal in the mouse orthologs of human genes known to exhibit R-loops^23,24^ further confirmed the quality of our DRIP-seq analysis and revealed that R-loop formation in these genes is conserved between mice and humans and across cell types (Fig. S1C). Analysis of NSPC DRIP-seq libraries generated from nine DRIP samples prepared from three independent, biological replicates (i.e., three technical repeats per each of the three biological replicates) revealed a total of 22,132 R-loop peaks. R-loop peaks covered 1.01 ± 0.45% (mean ± S.D.) of the NSPC genome, which is similar to the extent of R-loop formation reported for other cell types and species^23^.

Further analysis revealed that R-loop peaks in NSPCs are significantly enriched in 5′-UTRs, promoters, introns, exons, transcription termination sites, and 3′-UTRs but are depleted in intergenic regions (Fig. 1C). Overall, NSPC R-loop peaks were detected in 9020 annotated genes (RefSeq NCBI37/mm9). Next, we assessed the transcriptional activity of genes containing R-loop peaks in NSPCs. Consistent with the notion that transcription promotes R-loop formation, the vast majority (99.17%) of genes containing R-loop peaks was either transcriptionally active (GRO-seq RPKM ≥ 0.025) or showed ambiguous (GRO-seq RPKM ≥ 0.0025 to < 0.025) transcriptional activity. Only 75 (0.83%) of the 9020 genes containing R-loop peaks in NSPCs were transcriptionally inactive (GRO-seq RPKM < 0.0025) (Fig. 1D).

To gain insights into the functions of R-loops and potential relationships to genomic stability in NSPCs undergoing mild replication stress, we further assessed the genomic distribution of DRIP-seq reads. DRIP-seq signal in NSPCs was present throughout the gene bodies of actively transcribed genes and showed a robust enrichment around the transcription start sites (TSSs) and transcription end sites (TESs) of active genes (Fig. 1E). These findings are consistent with the reported distribution of R-loops in other cell types and their involvement in regulatory functions in these regions^24–29^ and reveal that this distribution persists under mild replication stress in NSPCs.

### Comparative analysis of R-loop formation in NSPCs and embryonic stem cells

To compare our DRIP-seq results from NSPCs with published DRIP-seq data and gain insights into potential, lineage-specific features of R-loop formation, we obtained the deposited FASTQ files from DRIP-seq studies in pluripotent, mouse embryonic stem cells (ESCs)^23^. To enable direct comparisons, we performed all data analysis of NSPC and ESC DRIP-seq under identical bioinformatic conditions. R-loop peaks in aphidicolin-treated NSPCs and untreated ESCs showed a similar distribution across chromosomes (Fig. S2A) and displayed a similar mean R-loop peak size of around 2 kb (NSPC, 2.19 ± 0.05 kb; ESC, 1.95 ± 0.02 kb; mean ± S.E.M) (Fig. S2B). Overall, the ESC DRIP-seq data set contained a slightly higher total number of R-loop peaks (57,751) than detected in the combined NSPC DRIP-seq data, but when normalized via random down-sampling to the total peak number observed in NSPCs, ESCs and NSPCs showed similar absolute R-loop peak numbers and R-loop densities across chromosomes (Fig. S2C and D). As in aphidicolin-treated NSPCs, analysis of R-loop peak distribution in untreated ESCs revealed enrichment in 5′-UTRs, promoters, introns, exons, transcription termination sites, and 3′-UTRs, and depletion in intergenic regions (Fig. S2E).

**Figure 2:**
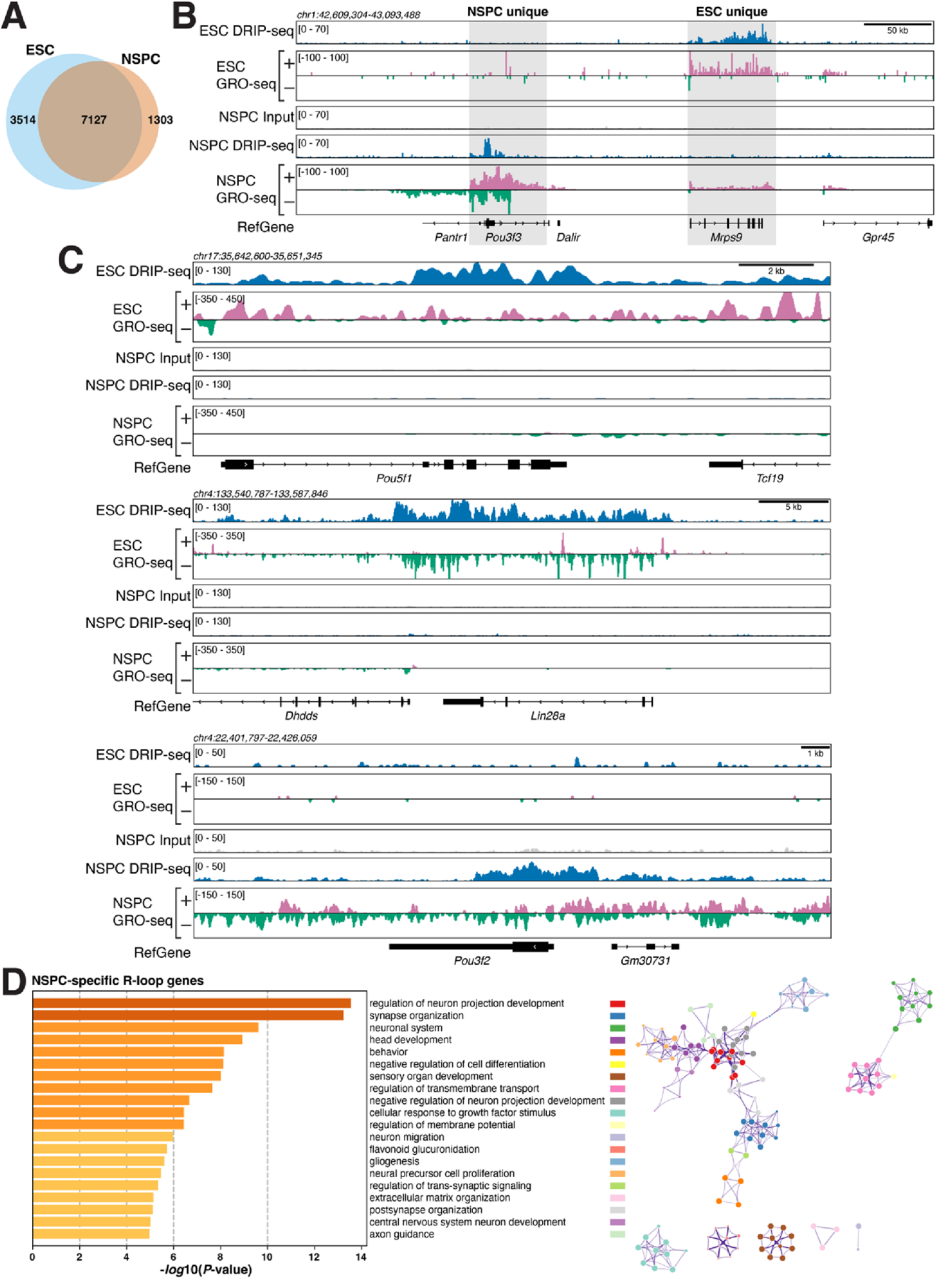
The landscape of R-loop formation in NSPCs suggests roles in lineage-specific processes. (**A**) Venn diagram showing the number of common and unique genes with R-loop peaks in ESCs and NSPCs under mild replication stress. (**B**) Examples of genes forming R-loops in NSPCs or in ESCs. RPKM-normalized DRIP-seq and GRO-seq signals and RefSeq genes are shown. (**C**) Lineage-specific genes form R-loops in ESCs and NSPCs, illustrated as in (**B**). (**D**) *Left*, gene ontology (GO) analysis of NSPC-specific R-loop genes. Bars show significantly enriched GO terms and are colored by *P* values in *log* base 10. The Top 20 clusters are shown. *Right*, network visualization of the enriched terms shown on left, colored by cluster ID. Nodes sharing the same cluster ID are close to each other.

Next, we asked if R-loops in NSPCs are associated with genes involved in specific cellular functions and processes. To this end, we determined which genes show ≥ 1 R-loop peak in both ESCs and NSPCs ("common"), or ≥ 1 R-loop peak uniquely in either cell type ("ESC unique"; "NSPC unique") (Fig. 2A). The group of "common" R-loop genes contained 7127 genes, representing 66.98% and 84.54% of active genes with R-loops in ESC and NSPCs, respectively. 1303 genes (15.46%) were unique to NSPCs, and 3514 genes (33.02%) were unique to ESCs (Fig. 2A). Figure 2B shows examples, with *mitochondrial ribosomal protein 9* (*MrpS9*) being actively transcribed and forming R-loops in ESCs and *Pou3f3* (also known as *Brain-1*), a gene with roles in brain development^30^ and intellectual disability^31^ being unique to NSPCs. Core ESC transcriptional factors such as *Pou5f1* and *Lin28A* were unique to ESCs (Fig. 2C). *Pou3f2* (*Brain-2*), a gene involved in the establishment of neural cell lineage, neocortical development and associated with psychiatric disorders^32,33^ was unique to NSPCs (Fig. 2C). Notably *Pou3f3/Brain-1* acts synergistically with *Sox11* and *Sox4* in neural development and we find that both show robust R-loop formation in NSPCs (Fig. S3A). Moreover, R-loops in *Pou3f3/Brain-1* extended into the neighboring *Pantr1* (*Pou3f3 adjacent non-coding transcript 1*) gene, which encodes a long non-coding RNA implicated in glioma development^34^.

To assess the overall implications of R-loop formation within genes in the common, ESC- unique, and NSPC-unique sets, we performed pathway and process enrichment analyses (Figs. 2D and S3B-C). Strikingly, we found that genes with unique R-loop formation in NSPCs were significantly enriched in processes related to neural development and function (Fig. 2D). In stark contrast, shared R-loop genes were enriched for general biological processes (Fig. S3B) and genes in the ESC-specific set showed enrichment of more general cellular processes, including DNA repair, cell cycle, and DNA replication (Fig. S3C). Given the association between transcription and R-loop formation, we expected that similar results would be observed when considering genes just based on their transcriptional activity, *i.e.*, regardless of R-loop status. Analysis of GRO-seq data revealed 778 genes with unique, active transcription (GRO-seq RPKM ≥ 0.025) in NSPCs (Fig. S3D). This set of genes showed enrichment for processes related to neural development and function but less so than the set of genes with R-loops unique to NSPCs, with fewer terms clearly related to neural function and development (Figs. 2D and S3E).

Most of the differences in R-loop peaks between the two cell types are likely due to differences in transcription (Fig. 2B,C). However, some genes with similar rates of transcription in both NSPCs and ESCs show strikingly different levels of R-loops (Fig. S4). Although beyond the scope of our current study, it will be informative to elucidate why these genes show a decoupling of R-loop formation and rate of transcription. Specifically, 1034 of 1303 (79.36%) genes with R-loops only in NSPCs show a higher transcription rate in NSPCs than in ESCs. 35 genes (2.69%) of genes with NSPC-specific R-loop peaks are transcribed at similar levels (± 5% transcriptional activity as measured by GRO-seq RPKM) in ESCs, and 234 (17.96%) genes with R-loop peaks in NSPCs showed higher transcription, but no R-loop peaks, in ESCs. Similarly, of the 3514 genes with R-loop peaks specific to ESCs, 957 (27.23%) show higher transcription in NSPCs, 165 (4.70%) display similar rates of transcription in both cell lineages, and most genes with ESC-specific R-loop peaks (2392; 68.07%) show higher transcriptional activity in ESCs.

Overall, our comparative analysis of R-loop signal in NSPCs and ESCs points to potential, lineage-specific functions of R-loops and suggests that perturbation of R-loop formation in NSPCs may impact neural processes and development, given the association between R-loop formation and transcription of cell type-specific genes.

### Factors associated with R-loop formation

**Figure 3:**
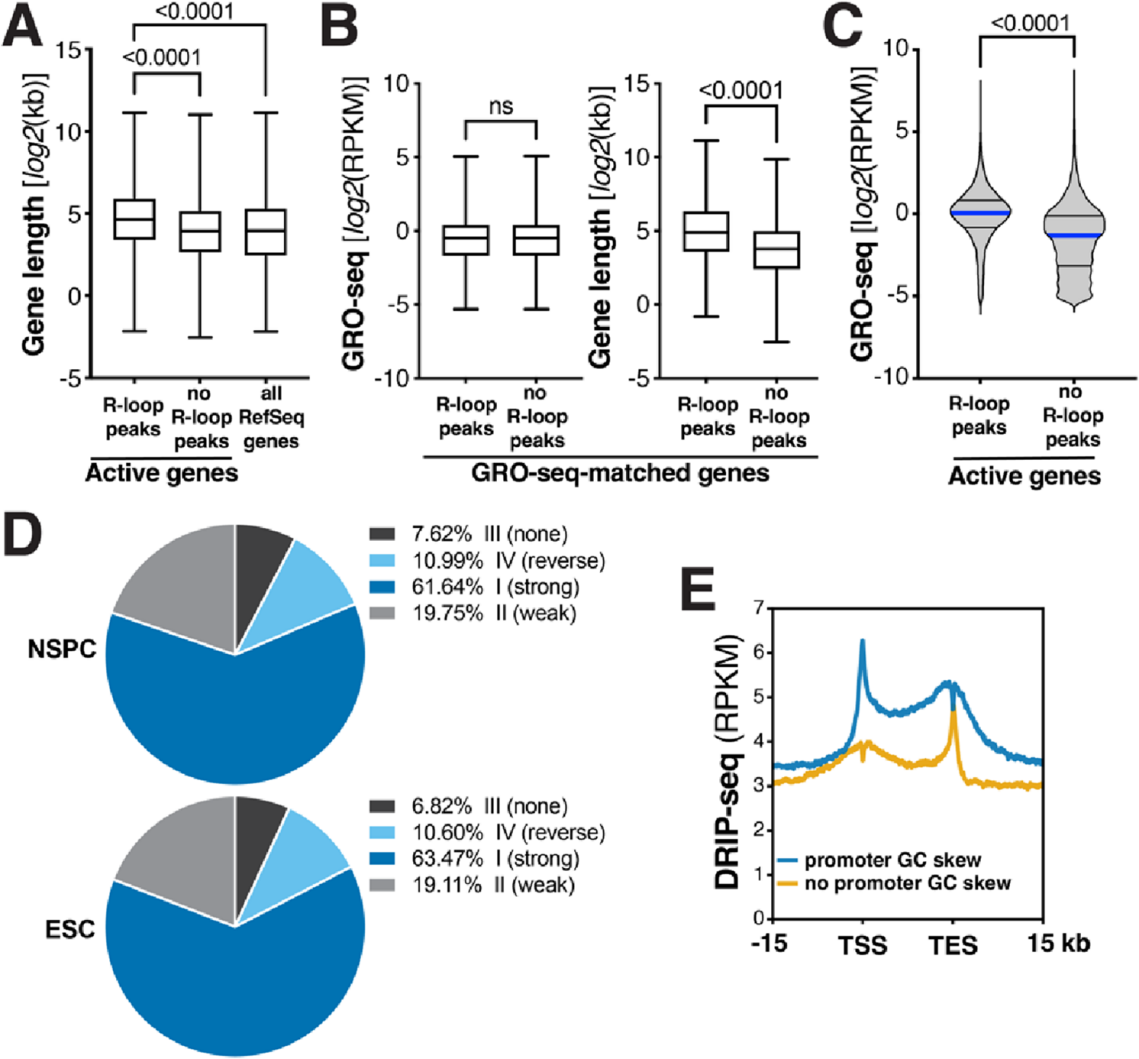
Factors promoting R-loop formation in NSPCs. (**A**) Box-and-whiskers plot showing gene length of active genes with (*n* = 8430) or without (*n* = 7093) R-loop peaks in NSPCs and all RefSeq genes (*n* = 22,735), for comparison. *P* values were determined by one-way ANOVA with Tukey’s post hoc correction. (**B**) Comparison of gene length of transcription rate-matched genes (*n* = 3255) with or without R-loop peaks in NSPCs (*P* < 0.0001; *ns,* not significant; Mann–Whitney U test). (**C**) Transcriptional activity of active genes with (*n* = 8430) or without (*n* = 7093) R-loops in NSPCs. *P* < 0.0001, Mann–Whitney U test. (**D**) Pie charts showing distribution of genes with R-loop peaks in NSPCs (*top*) or ESCs (*bottom*) across the indicated classes of GC skew, showing that most genes with R-loops exhibit GC skew. (**E**) Metaplot analysis of RPKM-normalized raw DRIP-seq signal across the TSS, gene body, and TES of active NSPC genes with (blue, *n* = 7079) or without GC skew (yellow, *n* = 8449) in the promoter region.

Active genes containing R-loop peaks in NSPCs were significantly longer than active genes without R-loops, with an average gene length of 62.68 ± 1.39 kb (S.E.M.) and 38.7 ± 0.98 kb (S.E.M.), respectively (Fig. 3A). Notably, this difference in length persisted when we only compared genes with or without R-loops that showed a similar rate of transcription (Fig. 3B). Consistent with this observation, R-loop peak-containing genes in ESCs were longer than genes without R-loops (54.58 ± 0.98 kb vs. 36 ± 1.26 kb; mean ±  S.E.M.; Fig. S5A) and, again, this length difference persisted when comparing only transcription rate-matched genes with or without R-loops in ESCs (Fig. S5B). As a group, active genes with R-loop peaks showed a significantly higher level of transcription than active genes without R-loop peaks in both NSPCs undergoing mild replication stress (Fig. 3C) and ESCs under basal conditions (Fig. S5C). These findings indicate that in both NSPCs and ESCs, R-loop-forming genes are generally longer and more actively transcribed than genes without R-loops.

For reasons we do not currently understand, genes with R-loop peaks unique to NSPCs were on average significantly longer than genes with R-loops common to both cell types and genes with R-loop peaks unique to ESCs (Fig. S5D) and showed a significantly lower rate of transcription than either group of genes with R-loops (Fig. S5D). To assess this further, we compared the length of all actively transcribed genes (GRO-seq RPKM ≥ 0.025) in NSPCs (*n* = 15,528) and ESCs (*n* = 16,683). We found that actively transcribed genes in NSPCs are, on average, longer than actively transcribed genes in ESCs (51.72 ± 0.88 kb vs. 47.85 ± 0.77 kb; mean ±  S.E.M; *P* < 0.01, Mann–Whitney U test). Thus, one factor contributing to the greater length of genes with NSPC-specific R-loops may be that long genes are an expression feature of neural cells^35^.

To further assess factors contributing to R-loop formation in NSPCs, we used a 4-state hidden-Markov model (StochHMM)^25,26^ to predict GC skew regions in the mouse genome. After identifying regions with GC skew, R-loop peak-containing genes in NSPCs were clustered into four skew classes (strong skew, weak skew, no skew, and reverse skew)^25,26^. This analysis revealed that most R-loop-forming genes show GC skew, with only a minority (7.62%) exhibiting no GC skew (Fig. 3D, *Top*). Genes with R-loop peaks in ESCs showed a similar distribution across the four skew classes (Fig. 3D, *Bottom*), suggesting that—regardless of replication stress and cell type—GC skew is a universal feature of R-loop-forming genes, which is also supported by findings in human cells^25^.

To determine the impact of GC skew within the promoter region (± 2 kb of TSS) on R-loop formation in NSPCs, we divided all 15,528 genes that are actively transcribed in NSPCs into two groups; one group contained genes with GC skew within the promoter region (7079 genes), whereas the other group contained genes without GC skew within the promoter region (8449 genes). We then plotted the reads per kilobase per million (RPKM)-normalized DRIP-seq signal over these two groups of genes. Strikingly, genes with GC skew within 2 kb of the TSS showed a stronger DRIP-seq signal at the TSS and over the entire gene body and TES than genes without GC skew within the promoter region (Fig. 3E). In contrast, the latter group of genes showed a robust peak at the TES (Fig. 3E). We do not know why genes without TSS-proximal GC skew show extensive R-loop signal at the TES. One potential explanation may be that these genes rely more heavily on R-loop-mediated RNAPII pausing at their 3′ end^36^.

Together, our results reveal that gene length and rate of transcription are factors associated with R-loop formation and that GC skew in the promoter region is a strong predictor of overall R-loop formation throughout genes in NSPCs.

### Interplay between R-loop formation and DNA breakpoints in NSPCs

**Figure 4:**
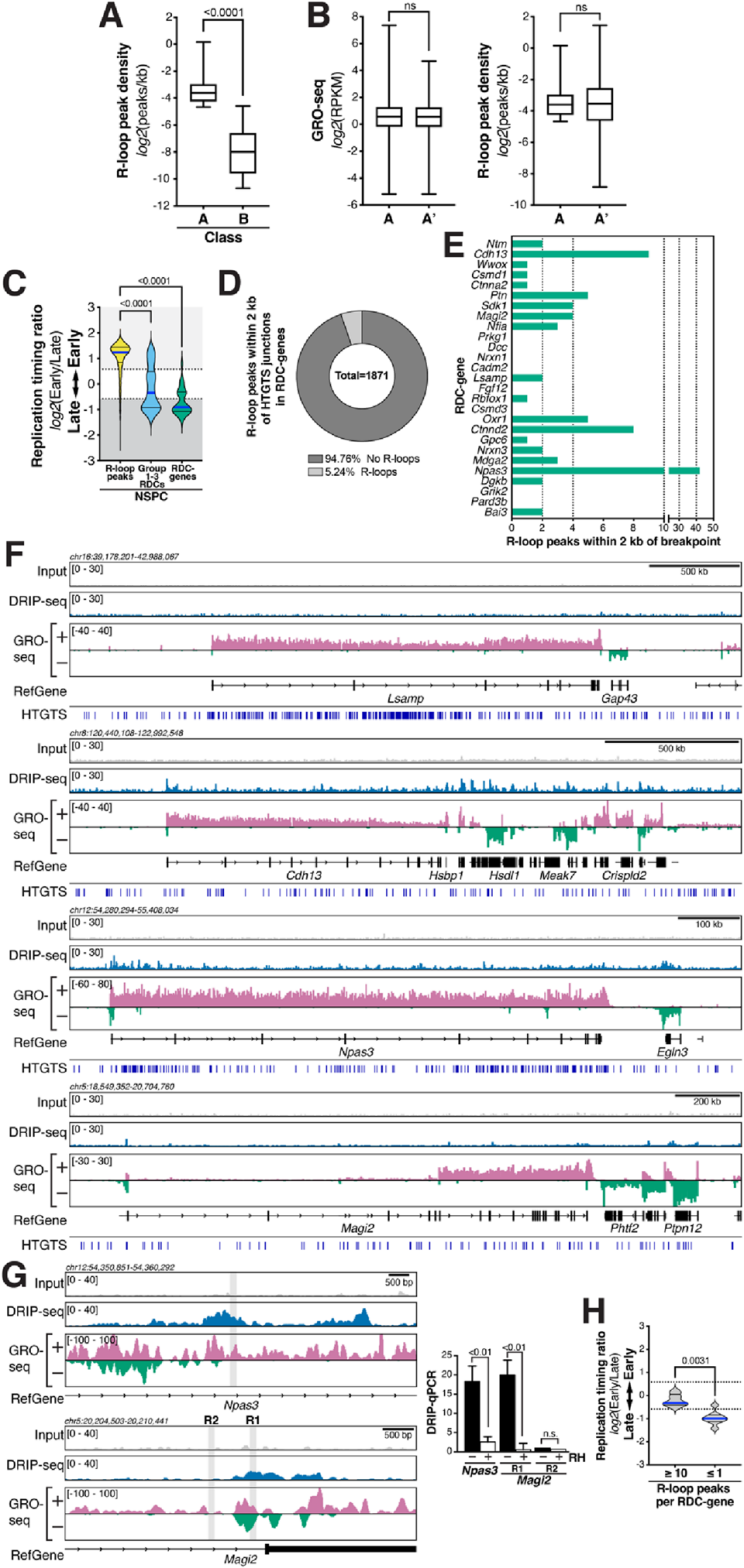
R-loops form in early-replicating regions with TSS-proximal DSBs, but RDC-genes are not prone to R-loop formation in NSPCs undergoing mild replication stress. (**A**) NSPC genes with TSS-proximal (± 2 kb) breakpoint junctions ("A", *n* = 382) show a significantly higher R-loop peak density than RDC-genes with R-loop peaks ("B", *n* = 23) overall. Whiskers show minimum and maximum values; top and bottom edges of boxplots correspond to 25th and 75th percentiles, respectively; horizontal lines indicate the median. *P* value was determined by the Mann–Whitney U test. (**B**) *Left*, Transcriptional activity of class A (*n* = 382) and transcription-matched gene set A' (*n* = 367); *Right*, Box-and-whisker plots of R-loop peak density in the two groups. Plot details are as in (A). (**C**) Violin plots showing the frequency distribution of replication timing ratios of all R-loop peaks (*n* = 22,132), Group 1–3 RDCs (*n* = 113), and the set of 27 RDC-genes in NSPCs. Median (blue line) and quartile lines (black) are shown. *P* values were determined by one-way ANOVA with Tukey’s post hoc correction. (**D**) Parts-of-whole graph showing fraction of RDC-gene HTGTS junctions that fall within two kb of an R-loop peak. (**E**) Bar graphs indicating numbers of R-loop peaks within two kb of a breakpoint junction in RDC-genes in NSPCs. (**F**) RPKM-normalized DRIP-seq signal over the indicated RDC-genes. Combined signal from DRIP samples and matching input controls from three biological replicates of aphidicolin-treated NSPCs is plotted. RPKM-normalized NSPC GRO-seq signal is shown to indicate transcription. RefSeq genes are shown in black. DSB junctions detected in NSPCs via HTGTS are indicated. (**G**) *Left*, zoomed-in visualization of DRIP-seq signal in RDC-genes *Npas3* (*top*) and *Magi2* (*bottom*), as in (**F**). Grey rectangles indicate regions analyzed by DRIP-qPCR. *Right*, DRIP-qPCR analysis using primers located in the regions shown on left in *Npas3* and *Magi2* [with (R1) or without (R2, negative control) R-loop peak signal]. Where indicated, samples were treated with RNase H (RH) prior to DRIP. Treatment with RNase H significantly suppressed the DRIP-qPCR signal, consistent with R-loop formation in the tested regions. DRIP-qPCR signal intensity (mean ±  S.E.M) shows fold enrichment over the *Snrpn* negative control region. *P* values were determined by two-tailed, unpaired *t* test; ns, not significant. (**H**) RDC-genes with ≥ 10 R-loop peaks (*n* = 5) show a significantly earlier replication timing than RDC-genes with ≤ 1 R-loop peak (*n* = 8). Violin plots show the frequency distribution of replication timing ratios in the two subsets of RDC-genes. Median (blue line) and quartile lines (black) are shown. *P* value was determined by the Mann–Whitney U test.

We previously reported that breakpoint junctions are enriched around active TSSs in NSPCs^9^. To evaluate a potential role of R-loops in promoting this class of DSBs, we compared the gene length-normalized R-loop peak density of active genes of average length (i.e., 5.49–25.49 kb) containing an HTGTS junctions within two kb of the TSS ("Class A") and those of the most robust RDC-genes containing at least one R-loop peak ("Class B"), respectively (Fig. 4A). Actively transcribed NSPC genes of average gene length with TSS-proximal breakpoint junctions displayed a significantly higher R-loop peak density than RDC-genes (Fig. 4A). However, these results do not reveal whether R-loops or transcription per se contribute to the formation of TSS-proximal DSBs. To shed light on this, we compared the R-loop peak density of genes with TSS-proximal DSBs detected by HTGTS (Class A; see Fig. S6 for examples) with a set of genes matched for rate of transcription and containing at least one R-loop peak (Set A'; Fig. 4B). These sets of genes showed similar R-loop peak density (Fig. 4B), suggesting that R-loop de-regulation or processing rather than R-loop levels per se may be relevant for TSS-proximal DSB formation.

To further consider a potential involvement of R-loop formation in the various, recurrent classes of DSBs in NSPCs, we next examined the replication timing of R-loop peaks. R-loop peaks in NSPCs undergoing mild replication stress were present in early-replicating regions and showed, on average, a significantly earlier replication timing than Group 1–3 RDCs or the most robust 27 RDC-genes^6,7^ (Fig. 4C). These findings were corroborated in ESCs, where R-loop peaks showed a significantly earlier replication timing than the set of RDC candidates in ESCs^8^ (Fig. S7A). Notably, the replication timing of R-loop peaks in aphidicolin-treated NSPCs and untreated ESCs did not differ significantly (Fig. S7B). These findings suggest that R-loop peaks preferentially occur in early-replicating regions of the genome in these two types of stem cells.

Next, we examined the formation of R-loops in the 27 RDC-genes^6^. To this end, we determined the number of R-loop peaks within two kb of HTGTS breakpoint junctions (Fig. 4D). Strikingly, within the most robust 27 RDC-genes, only 98 out of 1871 breakpoint regions (5.24%) contained an R-loop peak, whereas the vast majority (94.76%) of all RDC-gene breakpoint regions did not show R-loop formation (Fig. 4D). To reveal potential differences in R-loop formation among the 27 RDC-genes, we determined the number of R-loop peaks within two kb of HTGTS breakpoints in each RDC-gene. Whereas some RDC-genes contained few to no R-loop peaks within two kb of an HTGTS junction, we noticed a range of R-loop formation, with *Npas3*, *Ctnnd2*, and *Cdh13* containing the most R-loop peaks (Fig. 4E,F). DRIP-qPCR analysis confirmed the formation of R-loops (Fig. 4G).

RDCs in long neural genes tend to occur in large introns. Given the extensive splicing of transcripts of RDC-genes such as *Nrxn1* and *Nrxn3*, we hypothesized that co-transcriptional splicing of such large introns may make these genes prone to R-loop formation via reannealing of the nascent transcripts to the DNA. To our surprise, however, neither *Nrxn1* nor *Nrxn3* showed extensive R-loop formation (Fig. 4E), suggesting that RDCs in these long neural genes are not associated with a propensity for R-loop formation, even in the presence of mild replication stress. In the latter context, we had hypothesized that late replication timing would promote R-loop formation via transcription/replication collisions. However, RDC-genes with the highest number of R-loop peaks showed significantly earlier replication timing than RDC-genes with the lowest number of R-loop peaks (Fig. 4H), suggesting that R-loops may contribute to DSBs in some RDC-genes. But surprisingly, we did not find abundant R-loop formation in the RDC-genes with the highest DSB density, indicating that R-loops are not a major driver of RDC formation in these long, late-replicating genes under mild replication stress conditions.

Overall, our investigation of R-loop distribution in NSPCs under the same mild replication stress conditions under which recurrent classes of DSBs have been identified supports the notion that TSS-associated DSBs and DSBs in the gene bodies of long, transcribed neural genes are caused by different mechanisms, with the former class potentially being affected by processes related to R-loop formation and processing, consistent with findings in other cell types^24,37,38^.

## Discussion

The primary goal of our study was to investigate the relationship between R-loop distribution and the recurrent classes of DSBs identified under conditions of mild replication stress in NSPCs. A limitation of our study is that we do not know which R-loops are present at baseline, *i.e*., in the absence of aphidicolin-induced replication stress, in NSPCs. It will clearly be of interest to perform further studies of the effects of replication stress and related genetic factors on the spectrum and distribution of R-loops.

Our findings demonstrate that under mild replication stress, primary NSPCs show R-loop enrichment at TSSs and TESs (Fig. 1E), consistent with a role of R-loops in regulation of gene expression and transcription termination^13,25,29,39^. We find that NSPCs undergoing mild replication stress share a common set of R-loop-containing genes with ESCs but also contain a substantial fraction of unique R-loop genes (Fig. 2A). These latter findings suggest potential cell lineage-specific role of R-loops. Indeed, we find that NSPC-specific R-loop genes are significantly enriched in genes with functions in neural development and neural function (Fig. 2D).

Further studies of R-loop biology in neural progenitors may reveal important insights into processes ranging from neurodevelopment to neurological diseases. Factors modulating R-loop formation may play roles in the generation of somatic alterations during neurodevelopment, which may affect the extent of somatic brain mosaicism and occurrence of brain disorders. In the latter context, mutations in R-loop processing factors cause neurological disease in humans. Based on our finding of R-loop formation in genes with neural functions, we speculate that R-loop-mediated neurological disorders may have a previously unanticipated neurodevelopmental etiology at the level of neural progenitors—for example by affecting genomic stability or function of epigenetic R-loop readers^39^. On a related note, DNA damage caused by augmented R-loop formation has been proposed as a unifying mechanism for myelodysplastic syndromes induced by splicing factor mutations^40^. Based on our work here, it is possible that splicing factor mutations promote neurological disorders via increased R-loop formation and DNA damage in neural progenitors. R-loops have been further suggested to promote the instability of pathogenic repeat sequences in trinucleotide repeat-associated neurological diseases such as Huntington's disease^41–44^. *Huntingtin* (*Htt*) can form R-loops when transcribed in vitro^41–44^. Our DRIP-seq analysis reveals that *Htt* forms R-loops in vivo in NSPCs (Fig. S8A), suggesting potential contributions of R-loops to Huntington's disease pathology.

Moreover, R-loops have recently been implicated in the etiology of *Embryonal Tumor with Multilayered Rosettes*, a malignant brain tumor almost exclusively affecting young children, via induction of DNA breaks^45^. Our findings that R-loops associate with DSBs in NSPCs may suggest a role in the etiology of brain tumors more broadly. Indeed, several of the R-loop-forming genes we identified in NSPCs show rearrangements and mutations in human low-grade and high-grade gliomas, including *Raf1*, *Daxx*, *Fgfr1*, *Lztr1*, *and H3F3A* (Fig. S8B)^46–48^, which warrants further studies of the role of R-loops in brain cancer development.

Several classes of recurrent DSBs have been discovered in neural progenitor cells^4,6,7,9^. However, the underlying mechanistic causes of these recurrent DSBs are unclear. We had hypothesized that R-loops contribute to RDC formation in long neural genes based on several considerations: (*1*) R-loops can form at sites of RNA polymerase pausing caused by transcription-replication machinery collisions^13,22,27^; (*2*) long neural genes that form RDCs undergo extensive co-transcriptional splicing and pre-mRNA processing^49^, which can induce DSBs via R-loop formation^50^; and (*3*) earlier work has implicated R-loop formation in common fragile site formation within a subset of large human genes, based on slot blot hybridization experiments in the *FHIT* locus^12^. Thus, we were surprised to find that R-loops do not preferentially and robustly form in RDCs-genes that undergo extensive splicing—such as *Nrxn1* and *Nrnx3*—nor in *Lsamp* (Fig. 4E,F), one of the most robust RDCs in mouse NSPCs and human cells^6,11^.

There are several, not mutually exclusive, potential explanations for our findings. R-loop formation in RDC-genes such as *Lsamp*, *Cadm2*, *Nrxn1*, *Csmd3*, and others may be highly dynamic and transient, thus making these R-loops difficult to capture in primary NSPCs. However, this seems unlikely as we detected robust R-loop signal in promoter regions and TESs where R-loops are known to assemble in a dynamic and transient manner. A more likely interpretation is that replication stress-induced RDC formation in long genes such as *Lsamp*, *Csmd3*, *Nrxn1*, *Nrxn3*, *Csmd3*, and others is not primarily driven by R-loop formation. This conclusion is further supported by the generally lower GC content of RDC-genes (Fig. S9). Moreover, our findings are consistent with recent work showing a paucity of R-loops in the center of large human genes and proposing that the determining factor of replication stress-induced genomic instability is transcription-dependent persistence of unreplicated DNA into mitosis rather than R-loop formation^51^.

We note, however, that there are differences in the extent of R-loop formation among the group of long RDC-genes in NSPCs. For example, the RDC-genes *Cdh1*3*, Npas3* and others (Fig. 4E,F) show varying degrees of R-loop formation, which may contribute to some DNA breakpoint formation in this subset of RDC-genes. Notably, this latter set of RDC-genes tends to show earlier replication timing than RDC-genes without R-loops (Fig. 4H). The observed differences in R-loop formation among RDC-genes in NSPCs may be due to differential enrichment of factors that affect the formation or removal of R-loops, which warrants further investigation. In the latter regard, very long neural genes are uniquely reliant on topoisomerase activity for transcription elongation^52^ and, intriguingly, Topoisomerase 1 (Top1) depletion results in R-loop gains in long, highly-transcribed genes that are anchored to Lamin B1 domains^38^. Moreover, Top1 inhibition decreases R-loop formation in early replicating regions in human cells^38^. Thus, in future studies, it will be important to test the role of Top1 inhibition on TSS-proximal DSBs, RDC-gene fragility, and R-loop formation in NSPCs.

Prior work indicated that the causes of TSS-proximal DSBs and DSBs in the gene bodies of long genes are likely distinct^1,6,9^. As a group, NSPC genes with breakpoint junctions within two kb of the TSS show significantly higher R-loop peak density than RDC-genes (Fig. 4A). This is consistent with the notion that transcription- and R-loop-associated processes may contribute to promoter-proximal DSBs in NSPCs. However, a set of genes with matched transcription rate shows similar R-loop peak density (Fig. 4B). Further work is required to fully elucidate the relationship between R-loops and TSS-proximal DSBs in NSPCs and to define the molecular factors mediating DSB formation in R-loop-prone regions in the NSPC genome. For example, DSBs can result from R-loop removal by transcription-coupled nucleotide excision repair^37^. In this regard, analysis of the role of factors such as the nucleotide excision repair endonucleases XPF and XPG in the formation of R-loop-associated DSBs in NSPCs will be of great interest^37^.

Finally, given the association we observe, both DNA damage-inducing and -protective roles of R-loops are possible. R-loops may, at least in some contexts, function as “beneficial” structures that help with DSB repair^14^. This notion is based on observations that R-loops form at DSB sites in response to various types of DNA damage, and DSB-induced R-loops may form in *cis* as a response to DSB-mediated repression of transcription^14^. Future work will need to address whether some of the R-loops we observe in the vicinity of DNA breakpoints have such roles in DSB repair in NSPCs, for example by recruiting repair factors such as Rad52.

## Methods

### Culture of primary NSPCs

NSPCs were isolated from frontal brains of postnatal day seven mice and cultured as described^6^. All experiments were authorized by the Institutional Animal Care and Use Committee and Institutional Biosafety Committee at the University of California, San Francisco (Protocol AN182936) and performed in accordance with relevant guidelines and regulations. The reporting in this manuscript follows the ARRIVE guidelines^53^. Where indicated, cells were treated with 0.5 μM aphidicolin (Sigma, A4487) for 48 h before processing for DRIP.

### DRIP analysis and genome-wide R-loop mapping by DRIP-seq

Genomic DNA for DRIP was isolated and digested with *EcoRI*, *HindIII*, *BsrgI*, *SspI*, *XbaI* (all from NEB) at 37 °C, as described^24,26^. RNase A treatment prior to DRIP was performed as described^22,23^. For Ribonuclease H (RNase H) treatment, 8 μg of DNA were treated with 30 U of RNase H (NEB, M0297) for 16 h at 37 °C. Digested DNA was phenol/chloroform-extracted, precipitated, washed and resuspended as described^24^. For each DRIP reaction, 4.4 μg DNA were incubated with 10 μg S9.6 antibody for 16 h at 4 °C^24^, followed by incubation with magnetic protein G beads (Dynabeads, ThermoFisher Scientific) for 2 h at 4 °C. Samples were washed for 3 × 10 min with 140 mM NaCl, 0.05% (w/v) Triton X-100, 10 mM NaPO4, pH 7.0, at room temperature and eluted in 10 mM EDTA, 0.5% (w/v) SDS, 50 mM Tris–Cl, pH 8.0 containing Proteinase K for 45 min at 55 °C^24^. DNA was phenol/chloroform-extracted and precipitated as described above. Per biological replicate, three repeat DRIP reactions were first analyzed separately by DRIP-qPCR to verify the DRIP procedure, as described^54^. Primers used for DRIP-qPCR analysis are listed in Table S1. Three DRIP reactions per biological replicate were then pooled for preparation of each DRIP-seq library. Input and DRIP DNA was sonicated (Diagenode Bioruptor) to a size of ~ 300–700 bp and DRIP-seq library preparation was performed as described^24,26^, using NEB E6050 for end repair; NEB M0212 for A-tailing, and NEB E7335 for adapter ligation. 12 cycles of PCR were performed for library amplification and libraries were cleaned and size selected (AMPure XP beads; A63880, Beckman Coulter) as described^54^. Libraries were assessed and quantified by using the Qubit HS assay (Invitrogen Thermo Scientific), Bioanalyzer High Sensitivity DNA Analysis (Agilent), and qPCR-based KAPA Library Quantification (Kapa Biosystems, KK4824). Pooled libraries were sequenced on the Illumina HiSeq next-generation sequencing platform.

### Anti-DNA:RNA hybrid S9.6 antibody

Hybridoma cells producing the monoclonal S9.6 antibody were obtained from ATCC (HB-8730; RRID:CVCL G144) and grown in chemically-defined, protein-free CD hybridoma medium (Gibco, 11279023). S9.6 antibodies were purified according to standard procedures^24^ by using a HiTrap Protein G HP column (GE Healthcare), followed by extensive washing with 20 column volumes of PBS and elution with five column volumes of elution buffer (0.1 M glycine–HCl, pH 2.7). Antibody-containing fractions were assessed for purity by SDS-PAGE and Coomassie blue staining and sequentially dialyzed against PBS and 50% (v/v) glycerol/PBS. Antibody concentration was adjusted to 1 mg/mL. Purified S9.6 antibodies were validated by dot blot and DRIP-qPCR analysis along with commercially available S9.6 antibodies (Kerafast, ENH001; S.H. Leppla, NIH).

### S9.6 dot blot analysis

DNA (5′-GTTCCCATATCCCGGACGAGCCC-3′) and RNA oligonucleotides (5′-rGrGrGrCrUrCrGrUrCrCrGrGrGrArUrArUrGrGrGrArArC-3′) were annealed in RNA:DNA hybridization buffer (20 mM NaCl, 10 mM Tris–HCl, pH 8.0) and spotted onto Nylon membranes (GE Healthcare, RPN303B). Membranes were dried, UV crosslinked (120 mJ/cm^2^), incubated in blocking buffer (10% (w/v) non-fat dry milk in TBS with 0.1% (w/v) Tween-20) and probed with 0.5 μg/mL S9.6 antibody in blocking buffer for 1 h at room temperature. After three washes in TBS with 0.1% (w/v) Tween-20, membranes were incubated with secondary anti-mouse-HRP antibodies in blocking buffer for 1 h at room temperature, washed again, developed (ECL), and exposed to film.

### Bioinformatic and statistical analysis

DRIP-seq and GRO-seq reads were adapter trimmed (TrimGalore 0.6.6; https://github.com/FelixKrueger/TrimGalore), aligned to the NCBI37/mm9 genome, and processed as described^6,23,54^. Mouse ESC GRO-seq and DRIP-seq FASTQ files were obtained via GEO^23,55^ and processed in parallel with NSPC GRO-seq^6^ and NSPC DRIP-seq data. Duplicate reads were removed during processing as described^54^. We used a Hidden Markov Model-based peak calling algorithm for identification of DRIP-seq peaks, exactly as described^23^. Normalized genome-wide densities of uniquely mapped reads were determined by deepTools2^56^. Reads per kb per million (RPKM)-normalized bigWig tracks were generated from BAM files containing uniquely mapped reads using deepTools2 and visualized in IGV^57^. Nucleotide content was analyzed by bedtools version 2.29.2^58^. Genome annotations were determined by HOMER version 4.11.1^59^ using HOMER mm9 v6.4 and mouse-o v6.3 accession and ontology information with '*annotatePeaks.pl* -annStats' and default settings. HTGTS breakpoint junction data was analyzed as described^6–9^. Median replication timing ratios were determined using Repli-chip data^60,61^ and custom Python scripts, as described^6^. Statistical analysis was performed in GraphPad Prism 9.2.0 and in R^62^.

### Identification of genomic regions with GC skew

We applied the two-phase SkewR pipeline 1.00b^25,26^ developed to define regions displaying GC skew in the human genome to the mouse NCBI37/mm9 genome. SkewR uses a four-state hidden-Markov model (StochHMM) to predict GC skew regions^25,26^. The algorithm involves training on regions from verified R-loop-forming regions in human and mouse regions. Regions displaying GC skew were identified and genes were clustered into four skew classes (strong skew, weak skew, no skew, and reverse skew) by using the most stringent threshold model file (GC_SKEW_1mil.hmm)^25,26^. For metagene plots, the GC_SKEW_7600 model file was used^25^.

### Pathway and process enrichment analysis of R-loop-containing genes

For each gene list, pathway and process enrichment was analyzed using the GO Biological Processes, KEGG Pathway, Reactome Gene Sets, CORUM, TRRUST, PaGenBase, WikiPathways and PANTHER Pathway ontology sources and Metascape version 3.5.202108015^63^. All mouse genes were used as the enrichment background. Enrichment terms with *P* < 0.01, at least three counts, and an enrichment factor (counts observed vs. counts expected by chance) of > 1.5 were identified and clustered based on their similarities. *P* values were calculated based on the accumulative hypergeometric distribution. *q*-values were calculated by the Benjamini–Hochberg procedure to account for multiple testing. Kappa scores were used as the similarity metric for hierarchical clustering of enriched terms and sub-trees showing > 0.3 similarity were considered a cluster, with the most statistically significant term chosen to represent the cluster^63^. Clusters were visualized by Cytoscape^64^.

## Supplementary Information

Supplementary Information.

## Data Availability

The datasets generated and analyzed during the current study are available in the NCBI Gene Expression Omnibus repository under accession numbers GSE195963, GSE74356, GSE142315, GSE27037, and GSE70189.
