# Supplementary material for: Relationships between genome-wide R-loop distribution and classes of recurrent DNA breaks in neural stem/progenitor cells
Source: Sci Rep. 2022 Aug 4;12:13373. doi: 10.1038/s41598-022-17452-0 (PMC9352722; doi:10.1038/s41598-022-17452-0)
Supplement: Supplementary file 1 — Supplementary Information. [file 41598_2022_17452_MOESM1_ESM.pdf]

Supplementary Information for

Relationships between genome-wide R-loop distribution and classes of recurrent DNA breaks in neural stem/progenitor cells

Supawat Thongthip, Annika Carlson, Magdalena P. Crossley, and Bjoern Schwer

Corresponding author:
Bjoern Schwer, UCSF Box 0520, San Francisco, CA 94143; Tel.: (415) 476-6786;
bjoern.schwer@ucsf.edu

SUPPLEMENTAL FIGURES AND TABLES

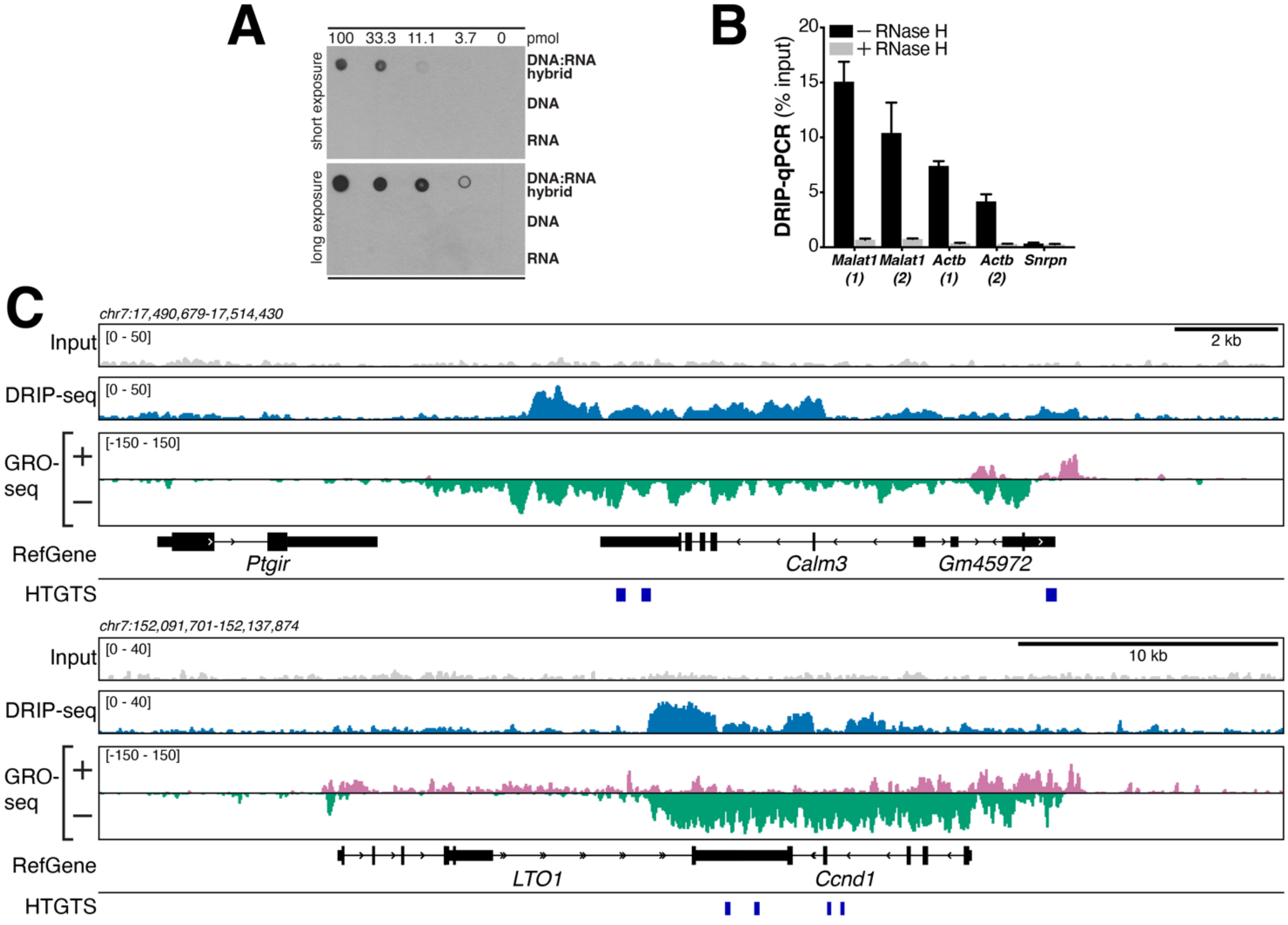

Fig. S1. DRIP-seq analysis of aphidicolin-treated NSPCs. (A) Dot blot analysis of serial dilutions of *in vitro* synthesized RNA-DNA hybrids (*top*) or equivalent amounts of the corresponding DNA (*middle*) or RNA (*bottom*) with the S9.6 anti-RNA-DNA hybrid antibody. **(B)** DRIP-qPCR analysis of the indicated positive and negative control regions in NSPCs. Two different sets of primers were used for *Malat1* and *Actb*, as indicated by the numbers below the graph. Bars show DRIP-qPCR signal as percentage of input. Pre-treatment with RNase H abolished the DRIP-qPCR signal. Data represent mean ± s.e.m. from three DRIP reactions performed in NSPCs. **(C)** RPKM-normalized NSPC DRIP-seq signal over the indicated genes, which also form R-loops in human cells[1,2]. Combined signal from nine DRIP samples and matching input controls corresponding to three biological replicates of APH-treated NSPCs is plotted. RPKM-normalized NSPC GRO-seq signal shows transcriptional activity. RefSeq genes are shown in black. DSB junctions detected by HTGTS in APH-treated NSPCs are indicated.

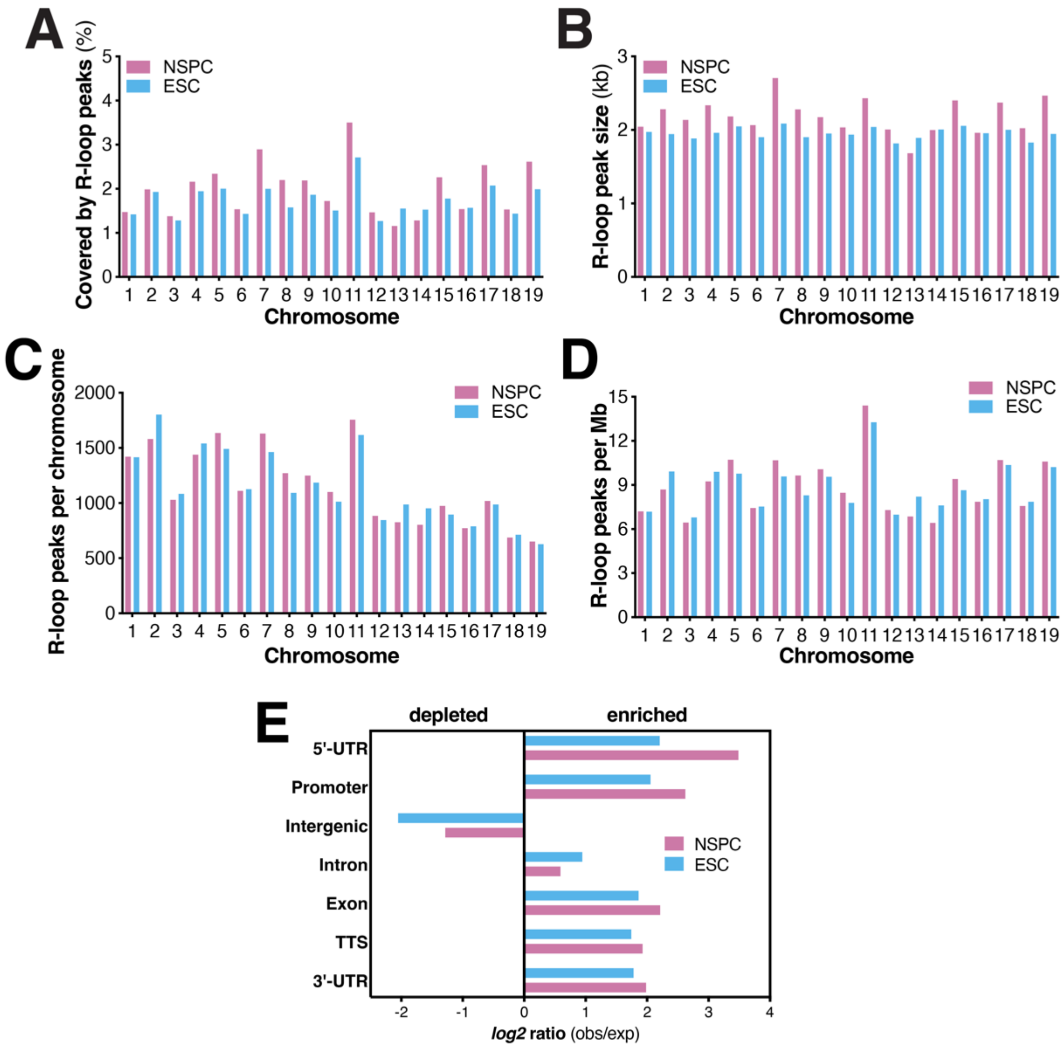

Fig. S2. Comparison of R-loop distribution and features in aphidicolin-treated NSPCs and untreated ESCs. **(A)** Coverage of the indicated chromosomes by R-loop peaks identified by custom Hidden Markov models[1] in NSPCs and ESCs. Bar charts show mean coverage per chromosome from three biological replicates of DRIP-seq performed in NSPCs. **(B)** Mean R-loop peak size in NSPCs (mean from three biological replicates) and ESCs. **(C)** Absolute numbers of R-loop peaks per indicated chromosome in NSPCs (mean from three biological replicates) and ESCs. **(D)** R-loop peak density in NPSCs (mean from three biological replicates) and ESCs. **(E)** Comparison of distribution of R-loop peaks across genome annotations in NSPCs and ESCs.

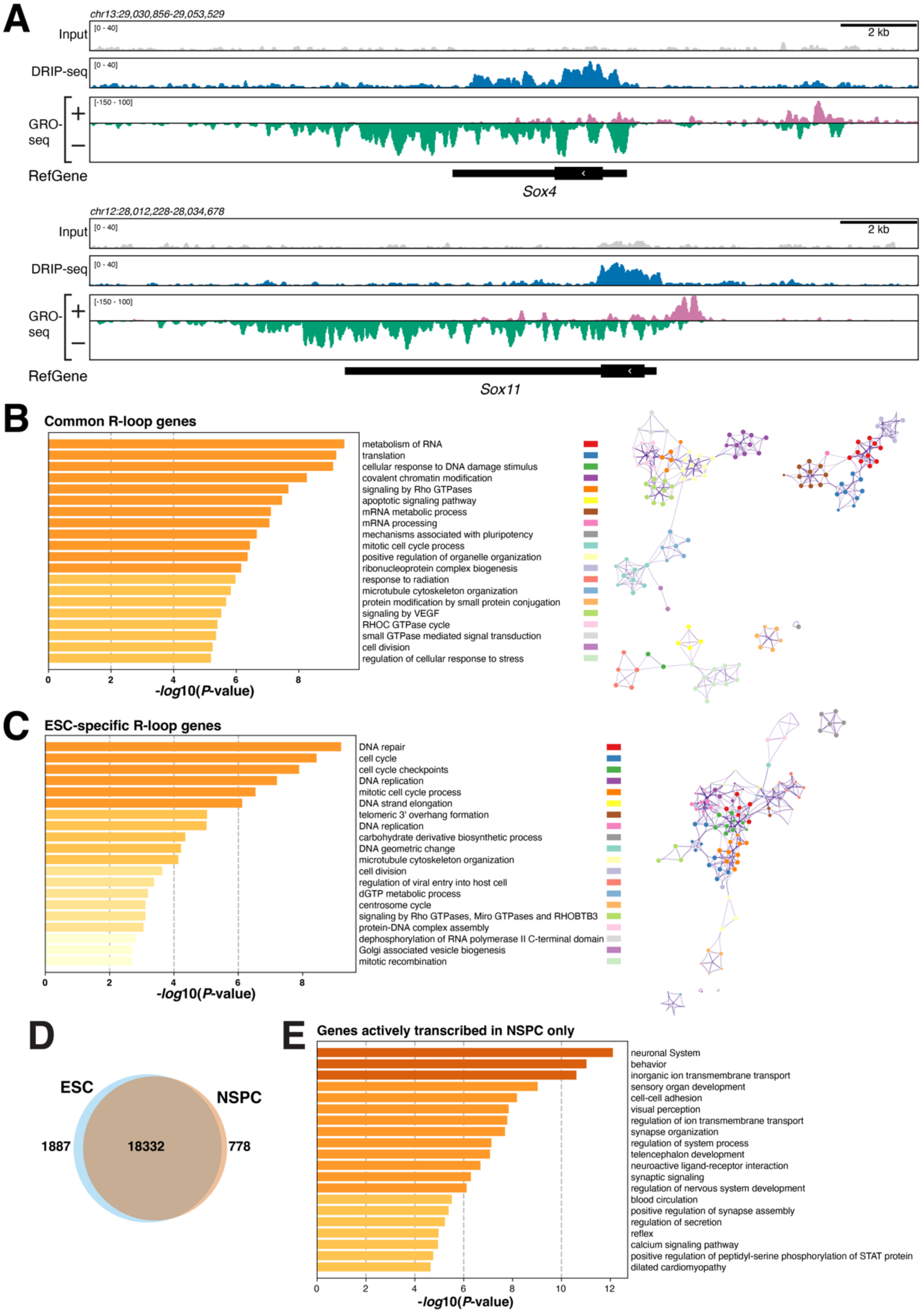

Fig. S3. R-loop formation in genes with general and lineage-specific functions in NSPCs and ESCs.
(A) R-loop formation in *Sox4* and *Sox11* in NSPCs. RPKM-normalized NSPC DRIP-seq and GRO-seq signal is shown. **(B)** *Left*, GO analysis of genes showing R-loop peaks in both NSPCs and ESCs. Bars show significantly enriched GO terms and are colored by *P* values in *log* base 10. The Top 20 clusters are shown. *Right*, network visualization of the enriched terms shown on left, colored by cluster ID. Nodes sharing the same cluster ID are close to each other. **(C)** GO analysis of genes showing R-loop peaks only in ESCs, as in (B). **(D)** Venn diagram showing the number of common and unique genes actively transcribed in ESCs and NSPCs. **(E)** Gene ontology (GO) analysis of genes uniquely transcribed NSPCs. Bars show significantly enriched GO terms, as in (C).

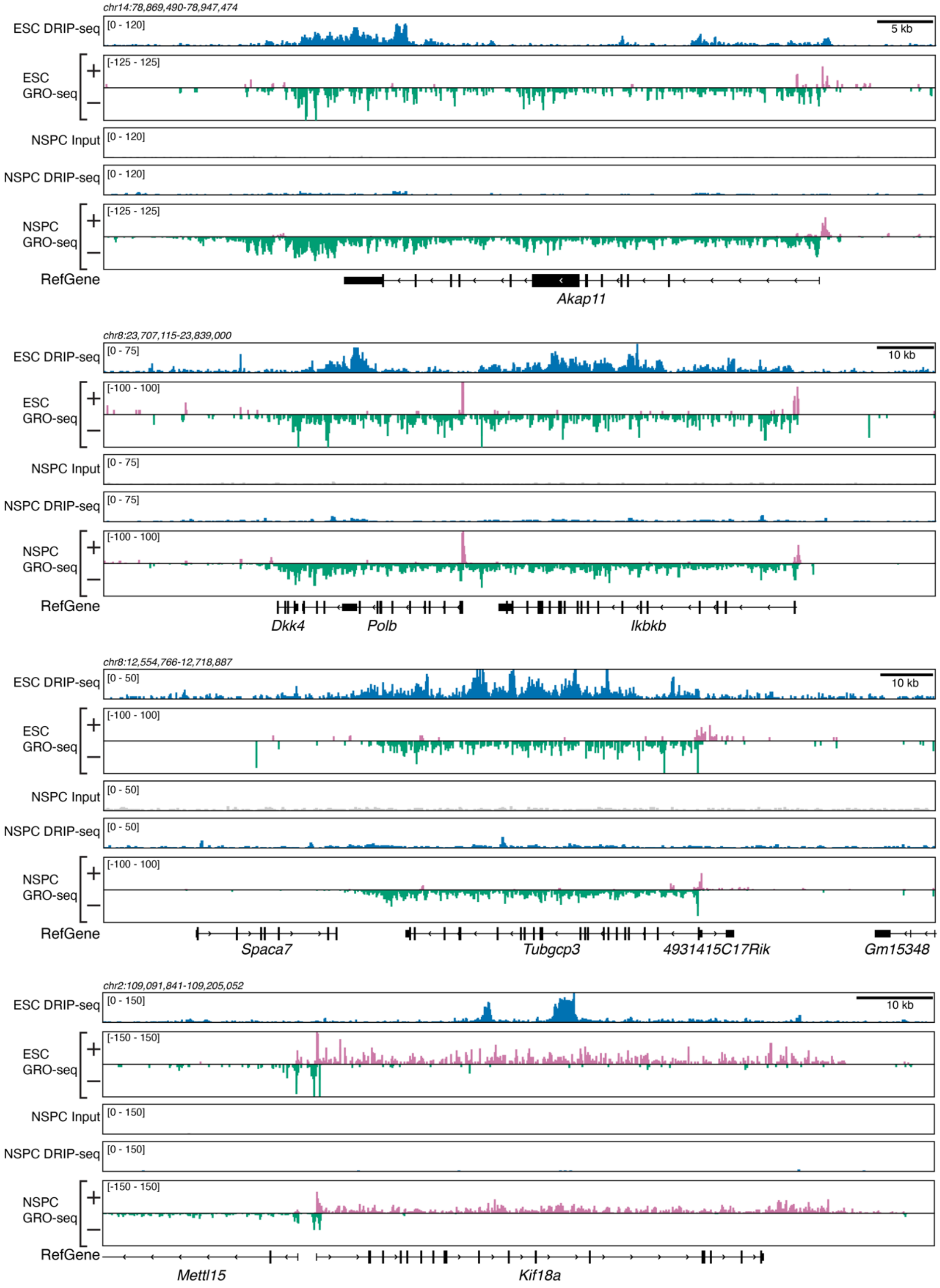

Fig. S4. Examples of genes with similar rates of transcription in both NSPCs and ESCs but strikingly different levels of R-loops. RPKM-normalized DRIP-seq and GRO-seq signals over the indicated genomic regions in ESCs and NSPCs. RefSeq genes are shown in black.

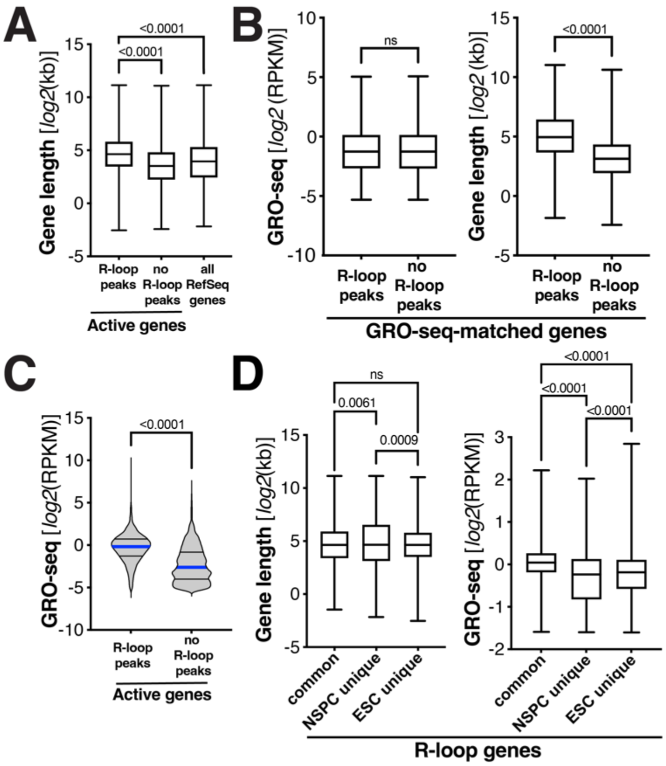

Fig. S5. Gene length and transcriptional activity of genes with or without R-loops in ESCs and NSPCs. **(A)** Box-and-whiskers plot showing gene length of active genes with (n=10,641) or without (n=6,042) R-loop peaks in ESCs and all RefSeq genes (n=22,735), for comparison. P-values were determined by one-way ANOVA with Tukey's *post hoc* correction. **(B)** Comparison of gene length of transcription rate-matched genes (n=2,883) with or without R-loop peaks in ESCs ($P < 0.0001$; *ns,* not significant; Mann-Whitney U test). **(C)** Transcriptional activity of active genes with (n=10,641) or without (n=6,042) R-loops in ESCs. $P < 0.0001$, Mann-Whitney U test. **(D)** *Left*, Length of genes with R-loops in both NSPCs and ESCs ("common", n=7,127), or only in NSPCs (n=1,303) or ESCs (n=3,514). *Right*, Transcriptional activity of the indicated groups of genes. P-values were determined by one-way ANOVA with Tukey's *post hoc* correction for multiple comparisons.

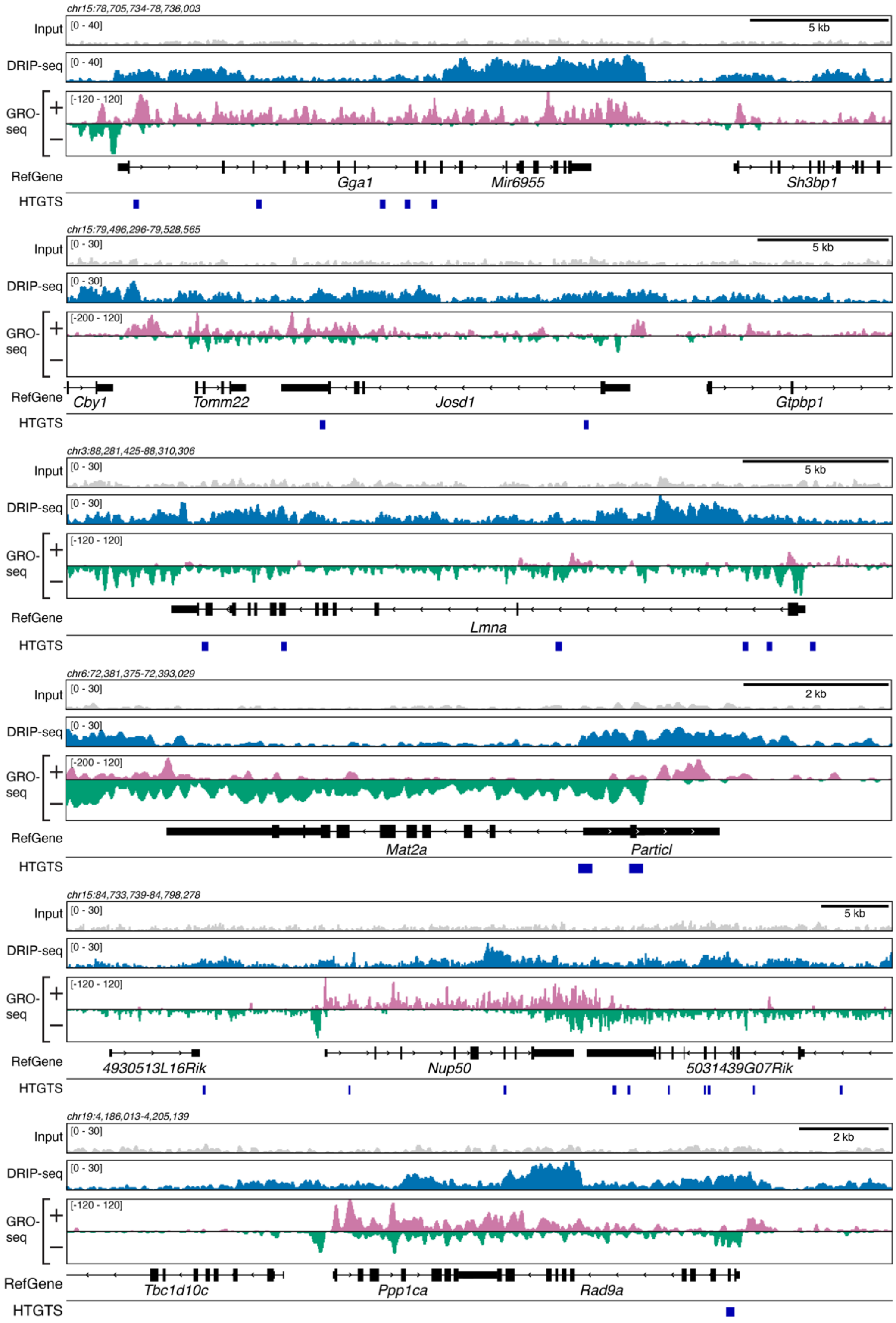

Fig. S6. Examples of genes with R-loops and TSS-proximal DSBs detected by HTGTS in NSPCs. RPKM-normalized DRIP-seq and GRO-seq signals over the indicated genomic regions in APH-treated NSPCs. RefSeq genes are shown in black. DSB junctions detected by HTGTS in APH-treated NSPCs are indicated.

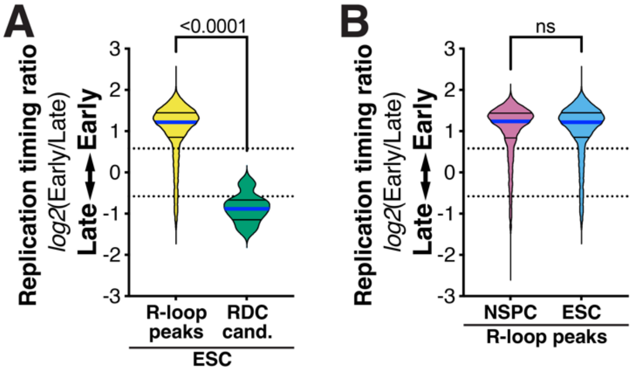

Fig. S7. Replication timing profiles and GC content of R-loop peaks and RDCs. (A) Violin plots showing the frequency distribution of replication timing ratios of all R-loop peaks (*n*=57,751) and the set of RDC candidates (*n*=7) in ESCs[3]. **(B)** Comparison of replication timing of all R-loop peaks in NSPCs (*n*=22,132) and ESCs (*n*=57,751). Median (blue line) and quartile lines (black) are shown. *P*-values were determined by the Mann-Whitney U test.

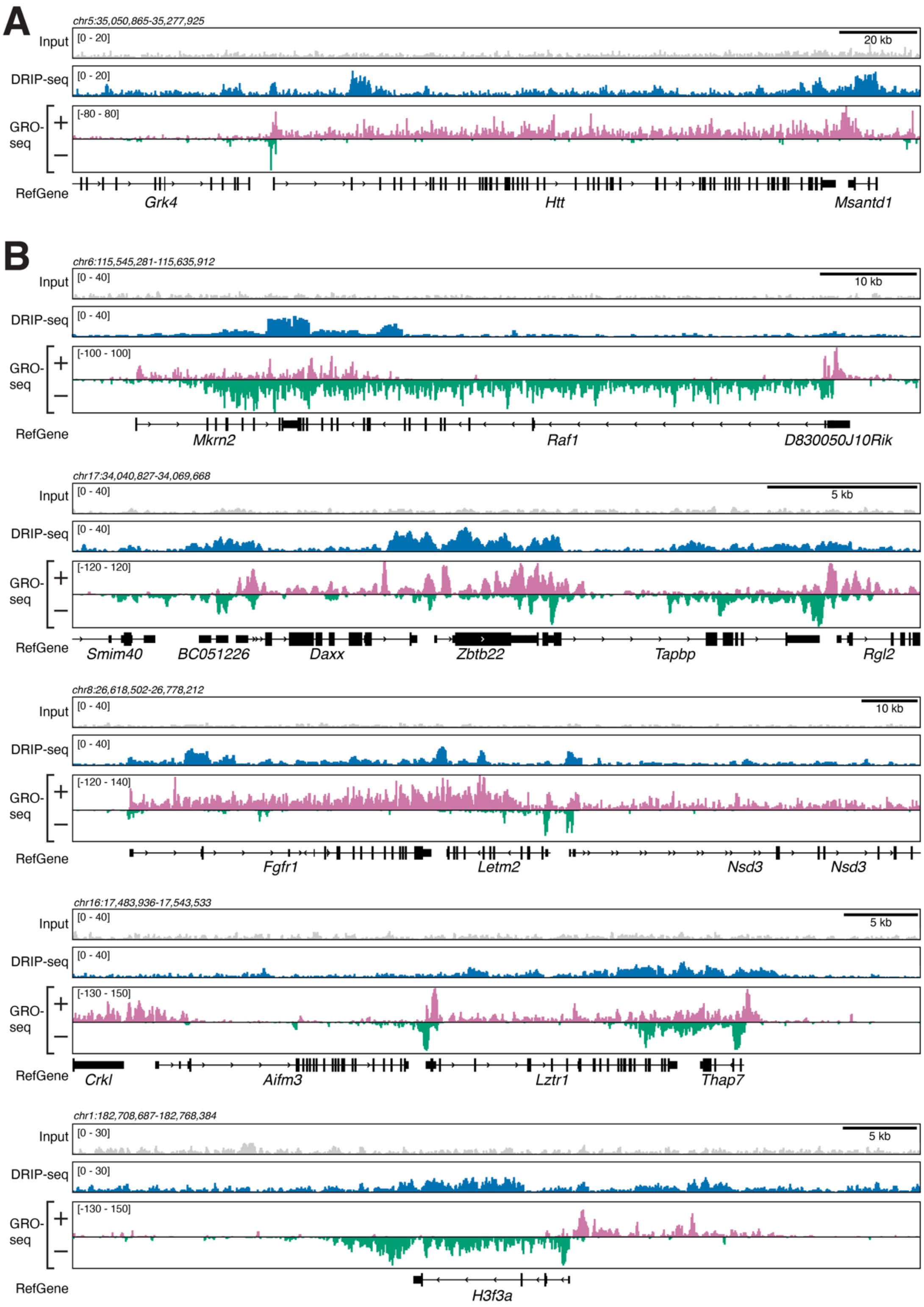

Fig. S8. NSPCs show R-loop formation in genes implicated in neurological diseases and brain cancer. **(A)** RPKM-normalized DRIP-seq signal over *Huntingtin* (*Htt*) and **(B)** genes with rearrangements and mutations in human low-grade and high-grade gliomas (*Raf1*, *Daxx*, *Fgfr1*, *Lztr1*, and *H3F3A*). Combined signal from nine DRIP samples and matching input controls from three biological replicates of APH-treated NSPCs is plotted. RPKM-normalized NSPC GRO-seq signal shows transcriptional activity. RefSeq genes are shown in black.

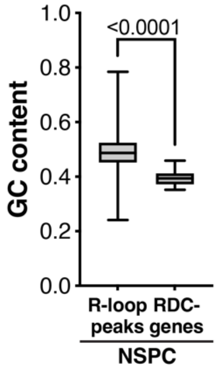

Fig. S9. GC content of R-loop peaks and RDCs. (C) Box-and-whiskers plot showing fractional GC content of all R-loop peaks (*n*=22,132) and RDC-genes (*n*=27) in NSPCs. Upper and lower box edges correspond to the 25th and 75th percentile; horizontal line indicates the median. *P*-value was determined by the Mann-Whitney U test.

Table S1. DRIP-qPCR primer sequences.

Name	Sequence
SnrpnF	5'-CAAAGACAGGACTCTGTGAACCT-3'
SnrpnR	5'-GGACCTAGTGCAGAATACTGTGG-3'
Npas3F	5'-GAGGAGTCATATGCCATCTAGTTTT-3'
Npas3R	5'-TCAGATGTGTGATTTTCTAGTTTCAA-3'
Magi2.R1F	5'-CATCCCAAGAATCGCAGAAG-3'
Magi2.R1R	5'-AGCAGGTCCCAGCAGTATAGAG-3'
Magi2.R2F	5'-GGGCGGACTCAAGATCACTA-3'
Magi2.R2R	5'-GGACACAAAGAAGCCACATTT-3'
Malat1.F1	5'-CGCAGTTGACAAGCCAAG-3'
Malat1.R1	5'-CAGGATGGTGGAGCGAGA-3'
Malat1.F2	5'-TCTTCTATTCTTCGGCTTCCTACT-3'
Malat1.R2	5'-AAGCATCTTTAGAAGACAGAAAAGGT-3'
Actb.F1	5'-CACGCGCAGCTAACTAGGA-3'
Actb.R1	5'-GCGTGCGCTCTCTATCACT-3'
Actb.F2	5'-CACGCGCAGCTAACTAGGA-3'
Actb.R2	5'-GCGTGCGCTCTCTATCACT-3'

SUPPLEMENTAL REFERENCES

1. Sanz, L. A. *et al.* Prevalent, dynamic, and conserved R-loop structures associate with specific epigenomic signatures in mammals. *Mol Cell* **63**, 167–178 (2016).
2. Stork, C. T. *et al.* Co-transcriptional R-loops are the main cause of estrogen-induced DNA damage. *Elife* **5**, e17548 (2016).
3. Tena, A. *et al.* Induction of recurrent break cluster genes in neural progenitor cells differentiated from embryonic stem cells in culture. *Proc Natl Acad Sci U S A* **117**, 10541–10546 (2020).
